# Exploring vascular contributions to cognitive impairment and dementia (ENIGMA): protocol for a prospective observational study

**DOI:** 10.1186/s12883-024-03601-7

**Published:** 2024-04-03

**Authors:** Sigrid Breinholt Vestergaard, Andreas Gammelgaard Damsbo, Niels Lech Pedersen, Katrine Zachariassen, Kim Ryun Drasbek, Leif Østergaard, Grethe Andersen, Rikke Beese Dalby, Janne Kærgård Mortensen

**Affiliations:** 1 Department of Neurology, Aarhus University Hospital, Palle Juul-Jensens Boulevard 99, Aarhus N, 8200 Denmark; 2 Department of Clinical Medicine, Aarhus University, Palle Juul-Jensens Boulevard 99, Aarhus N, 8200 Denmark; 3 Department of Neuroradiology, Aarhus University Hospital, Palle Juul-Jensens Boulevard 99, Aarhus N, 8200 Denmark; 4 Department of Clinical Medicine Center of Functionally Integrative Neuroscience, Aarhus University, Universitetsbyen 3, Aarhus C, 8000 Denmark; 5 Department of Radiology and Nuclear Medicine, University Hospital of Southern Denmark, Finsensgade 35, Esbjerg, 6700 Denmark; 6 Department of Clinical Medicine, Department of Neurology, Aarhus University, Aarhus University Hospital, Palle Juul-Jensens Boulevard 165, J109, Aarhus N, 8200 Denmark

**Keywords:** Stroke, Ischemic stroke, Cognition, Dementia, Small vessel disease, Capillary dysfunction, Plasma extracellular vesicles

## Abstract

**Background:**

Post-stroke cognitive impairment (PSCI) is common. However, the underlying pathophysiology remains largely unknown. Understanding the role of microvascular changes and finding markers that can predict PSCI, could be a first step towards better screening and management of PSCI. Capillary dysfunction is a pathological feature of cerebral small vessel disease and may play a role in the mechanisms underlying PSCI. Extracellular vesicles (EVs) are secreted from cells and may act as disease biomarkers. We aim to investigate the role of capillary dysfunction in PSCI and the associations between EV characteristics and cognitive function one year after acute ischemic stroke (AIS) and transient ischemic attack (TIA).

**Methods:**

The ENIGMA study is a single-centre prospective clinical observational study conducted at Aarhus University Hospital, Denmark. Consecutive patients with AIS and TIA are included and followed for one year with follow-up visits at three and 12 months. An MRI is performed at 24 h and 12 months follow-up. EV characteristics will be characterised from blood samples drawn at 24 h and three months follow-up. Cognitive function is assessed three and 12 months after AIS and TIA using the Repeatable Battery for the Assessment of Neuropsychological Status.

**Discussion:**

Using novel imaging and molecular biological techniques the ENIGMA study will provide new knowledge about the vascular contributions to cognitive decline and dementia.

**Trial registration:**

The study is retrospectively registered as an ongoing observational study at ClinicalTrials.gov with the identifier NCT06257823.

## Introduction and rationale

We continue to live longer, and the impact of age-related diseases such as stroke and dementia is growing [1]. In addition to the functional disability a stroke may cause, cognitive impairment after stroke is common. Post-stroke cognitive impairment (PSCI) is observed in up to 50% of stroke survivors, and a stroke doubles the risk of dementia [2–4]. Persistent cognitive impairment has also been found in patients with transient ischemic attack (TIA) [5], a disorder that is considered reversible by definition.

PSCI and dementia are associated with increased morbidity and mortality [6–8], however no specific disease modifying management exist. This has urged stakeholders to call for more research into the epidemiology, risk-factors, biomarkers, and mechanisms underlying PSCI [9, 10].

Many risk factors for PSCI have been identified including stroke severity, lesion size and location and common vascular risk factors such as age, hypertension, diabetes, atrial fibrillation, and smoking [5, 11–13]. Further, some magnetic resonance imaging (MRI) markers have been associated with PSCI including brain atrophy and markers of cerebral small vessel disease (cSVD) such as white matter hyperintensities and cerebral microbleeds [14, 15]. In addition, several blood-derived biomarkers have also shown promise as predictors of PSCI [16–18]. Despite these advances in the field, the exact underlying pathophysiology behind PSCI is not well understood. Identification of early pathologic changes associated with PSCI could be a first step in the development of a prognostic aid and in identifying future therapeutic targets.

Cerebral capillary dysfunction is characterized by limited oxygen extraction from the brain capillaries due to age- and risk factor-related capillary flow heterogeneity [19]. Capillary dysfunction is a pathophysiological feature of cSVD and may play an important role in the vascular mechanisms underlying PSCI [20, 21]. Advanced perfusion magnetic resonance imaging (MRI) techniques can detect and quantify capillary dysfunction and may help identify markers of PSCI [22–24].

Extracellular vesicles (EVs) are cell-derived membrane-enclosed vesicles (40-1000 nm diameter) secreted by most cell types including neurons and endothelial cells. They are involved in cell-to-cell communication and can cross the blood brain barrier [25]. The content, surface markers and release of EVs (hereafter called EV characteristics) are altered during disease processes. Because of their potential as diagnostic and prognostic biomarkers, EVs have recently become a field of interest [26]. Studies suggest that EV characteristics may change during acute stroke, in the chronic stroke phase and according to the level of cSVD [27–29]. However, associations between EV characteristics and post-stroke cognition are largely unstudied.

With this prospective clinical observational study, we aim to investigate the role of capillary dysfunction in PSCI and to examine the associations between EV characteristics and cognitive function after acute ischemic stroke (AIS) and TIA.

## Methods

### Design

The ENIGMA study is a single-centre prospective clinical observational study.

### Patient population

Patients aged ≥ 60 years admitted with AIS or TIA within 24 h of symptom onset and with a clinically relevant diffusion restriction identified on MRI diffusion weighted-imaging (DWI) are eligible for inclusion. Patients are enrolled from the comprehensive stroke center at Aarhus University Hospital in Denmark from April 2021 to June 2024, with last follow-up expected in June 2025. Detailed inclusion and exclusion criteria are summarized in Table 1.

A subset of patients participated in the clinical randomized controlled trial, Remote Ischemic Conditioning for Acute Stroke (RESIST) [30].

**Table 1:** Eligibility criteria

**Inclusion criteria**
• AIS or TIA with a clinically relevant diffusion restriction identified on MRI DWI-sequence on admission
• Admittance within 24 h from symptom onset
• Age ≥ 60
**Exclusion criteria**
• Dependency in activities of daily living (mRS score > 2)
• Known dementia, neurodegenerative disease, or other significant brain disease
• Concomitant life-threatening disease
• Contraindications to undergo MRI
• Allergy or intolerance to MRI contrast agents
• eGFR < 30
• Unable to give written informed consent
• Deemed unfit for follow-up

*AIS: acute ischemic stroke, TIA: transient ischemic attack, MRI: magnetic resonance imaging, DWI: diffusion-weighted imaging, mRS: modified Rankin scale, eGFR: estimated glomerular filtration rate*

### Protocol approvals and patient consent

The study is approved by the Danish regional research ethics committee (ID: VEK 1-10-72-253-20). The study is conducted according to the declaration of Helsinki. All patients provide written informed consent before enrollment.

### Study objectives

Primary objectives.

- To investigate associations between capillary dysfunction and PSCI.
- To investigate the associations between EV characteristics and PSCI.

Secondary objectives.

- To investigate associations between capillary dysfunction and functional outcome, well-being, and depression after stroke.
- To investigate the associations between EV characteristics and functional outcome, well-being, and depression after stroke.

### Study procedures and follow-up

**Fig. 1:**
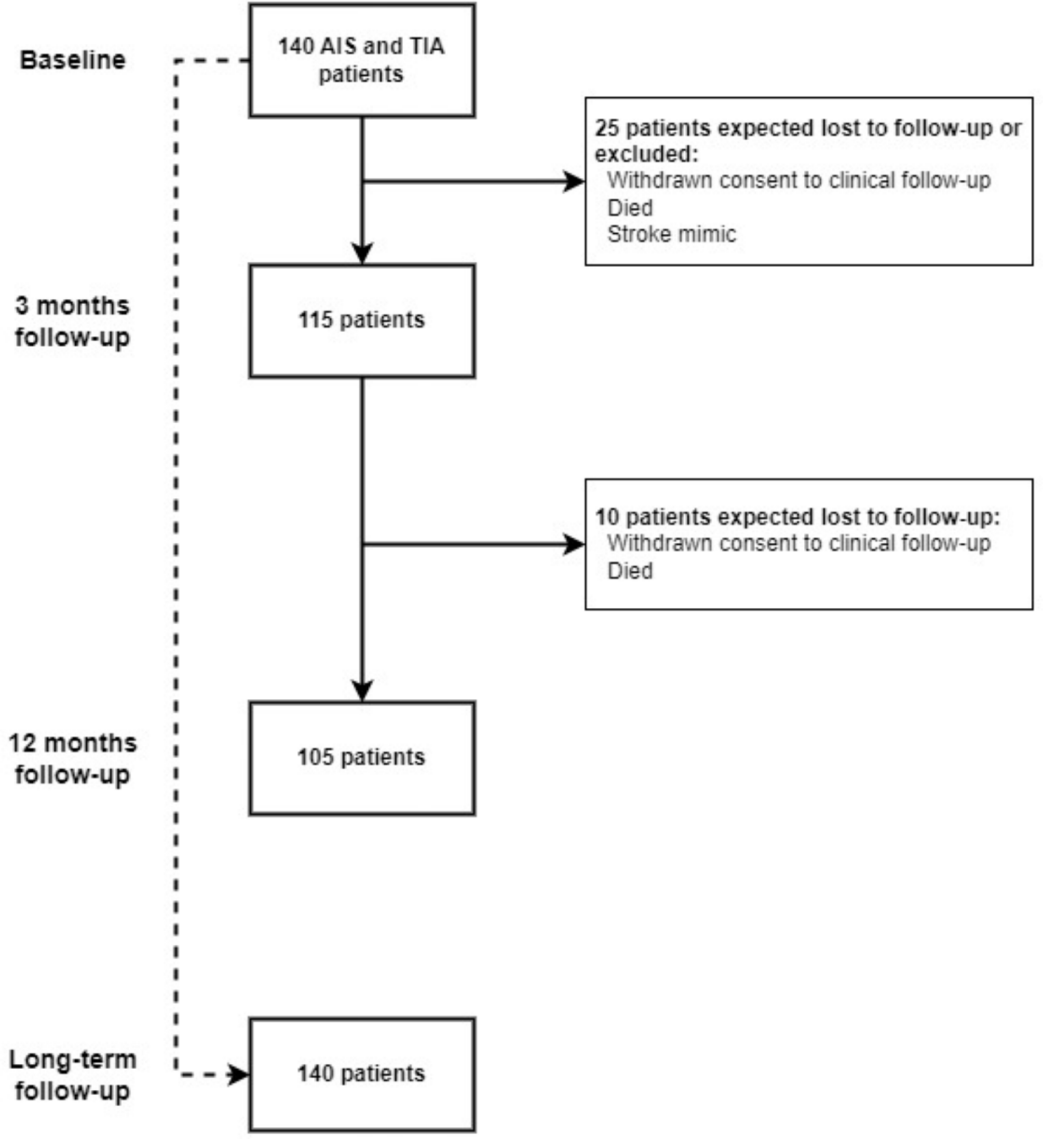
Anticipated inclusion and patient flow in the ENIGMA study

Eligible patients are included within the first 24 h of admission. Baseline measurements are carried out within 48 h from admission. In-person follow-up visits are conducted three months (+/- 1 week) and 12 months (+/- 2 weeks) after inclusion. An MRI is performed in extension to the 12-month follow-up visit. If patients decline to participate in the MRI or if they have incurred contraindications to undergo MRI, they are still eligible for the 12-month follow-up visit. In addition, long-term register-based follow-up is planned. It will include follow-up for recurrent stroke, other vascular events, vascular death, and dementia. Study procedures and their timings are listed in Table 2. Figure 1 illustrates anticipated patient inclusion and follow-up.

**Table 2:** Study assessments and registrations

Assessment/registration	Baseline	3-month follow-up	12-month follow-up	Long-term follow-up
Demographics	x			
Medical history	x			
Medication use	x			
Admission NIHSS score	x			
Symptom onset and admission time	x			
Acute treatment	x			
Standard blood chemistry at admission	x			
Pre-stroke mRS	x			
IQCODE	x			
Height, weight and BP	x	x	x	
Smoking and alcohol use	x			
MoCA	x	x	x	
PASE	x	x	x	
WHO-5	x	x	x	
MDI	x	x	x	
Educational level	x			
New vascular events		x	x	x
Functional outcome: mRS		x	x	
Stroke classification: TOAST and OSCP		x		
RBANS		x	x	
Blood sample for EVs*	x	x		
Blood sample for APOE genotyping		x		
MRI**	x		x	
Dementia				x

*Blood sample for EVs is drawn 24 h (+/- 8 h) after admission

**Baseline MRI is performed 24 h (+/- 6 h) after acute MRI on admission

*NIHSS: National Institutes of Health Stroke Scale, mRS: modified Rankin scale, IQCODE: Informant Questionnaire on Cognitive Decline in the Elderly, BP: blood pressure, MoCA: Montreal Cognitive Assessment, PASE: Physical Activity Scale for the Elderly, WHO-5: World Health Organisation Five Well-Being Index, MDI: Major Depression Inventory, TOAST: trial of Org 10,172 in Acute Stroke Treatment, OSCP: Oxfordshire Community Stroke Project Classification, RBANS: Repeatable Battery for the Assessment of Neuropsychological Status, EVs: extracellular vesicles, APOE: apolipoprotein E, MRI: magnetic resonance imaging*

### Magnetic resonance imaging

An MRI is performed at 24 h (+/- 6 h) after the acute MRI on admission and again at the 12-month follow-up visit. MRI sequences include T1-weighted 3D images for co-registration, DWI, kurtosis diffusion imaging, 3D fluid-attenuated inversion recovery (FLAIR), susceptibility weighted imaging (SWI), and gradient echo (GRE) and spin echo (SE) perfusion imaging.

White matter hyperintensities, visible on FLAIR images, are semi-automatically outlined [31]. Capillary function is characterized by capillary transit time heterogeneity (CTH), parameterized as the standard deviation of blood transit times within each image voxel using SE perfusion imaging [19, 22, 23]. Finally, grey and white matter microstructural integrity, including dendrite density, axonal density and dispersion, and intra- and extra-axonal water diffusivity are characterized based on DWI and kurtosis diffusion imagning [32–35]. All MRI scans are performed on site with a 3T Siemens Magnetom Vida scanner (Siemens Healthcare, Erlangen, Germany).

The burden of cSVD is assessed on the 24-hour and the 12-month MRI scans. A total cSVD score is calculated based on the STRIVE-1 criteria with local specifications [36]. The cSVD score includes number and location of microbleeds, superficial siderosis, number of lacunes, grade of white matter hyperintensities, grade of global cortical atrophy, and enlarged perivascular spaces. Scoring is independently performed by a trained neurologist and a neuroradiologist. At scoring disagreement, consensus is achieved. Table 3 presents an overview of the used MRI sequences.

**Table 3:** MRI sequences and characteristics at 24 h and 12 months

Sequence	Description	Purpose
DWI + ADC	Diffusion Weighted Imaging (DWI) and calculated Apparent Diffusion Coefficient (ADC)	Grey and white matter microstructural integrity, including dendrite density, axonal density and dispersion, and intra- and extra-axonal water diffusivity
Kurtosis	Kurtosis diffusion Imaging	
3D FLAIR	3-Dimensional Fluid-Attenuated Inversion Recovery	White matter hyperintensities, perivascular spaces, and lacunes
3D T1 (MP2RAGE)	3-Dimentional T1- magnetization Prepared Rapid Gradient Echo	Atrophy/cortical thickness
SWI	Susceptibility Weighted Imaging	Microbleeds and superficial siderosis
PWI GRE	Gradient Echo Perfusion Imaging	Capillary transit time heterogeneity
PWI SE	Spin Echo Perfusion Imaging	

## Blood samples, EV characterization, and genotyping

At 24 h (+/- 8 h) after admission and at the three-month follow-up visit, a blood sample consisting of three 3.5 mL citrate collection tubes is drawn by study personnel and centrifuged to yield plasma, which is stored at -80 °C. EV size and concentration will be determined after EV purification using size exclusion chromatography (Izon, Addington, New Zealand) and nanoparticle tracking analysis (NanoSight 300, Malvern Panalytica, Worcestershire, United Kingdom) [37]. Changes in EV surface markers will be studied using the EV Array platform that simultaneous assesses up to 60 EV surface markers [38]. In addition, changes in EV content (microRNA and proteins) will be assessed using nCounter microRNA panels (NanoString, Seattle, WA, USA) and Orbitrap mass spectrometers (Thermo Fisher Scientific, Waltham, MA, USA), respectively.

At the three-month visit, an additional blood sample in a 3 mL EDTA collection tube is drawn and stored at -80 °C without centrifugation. This full-blood sample will undergo DNA extraction and APOE genotyping.

### Cognitive assessments

Post-stroke cognitive function is assessed at three and 12-month follow-up visits using the Repeatable Battery for the Assessment of Neuropsychological Status (RBANS). To reduce learning bias, version A is administered at the three-month visit and version B is administered at the 12-month visit. All assessments are done by trained personnel. Training and scoring are overseen by a neuropsychologist and a neurologist specialized in dementia. All RBANS measures are administered and scored using standard procedures detailed in the Danish manual. The RBANS is a validated repeatable assessment of cognitive function [39, 40]. With 12 subtests it covers the five cognitive domains: immediate memory, visuospatial function, language, attention, and delayed memory. Based on norm data, the RBANS provides sub scores and age-adjusted index scores for each domain and combine them to a total index score.

The Montreal Cognitive Assessment (MoCA) is administered at baseline and at three- and 12-month follow-up. The MoCA is a brief cognitive screening tool validated for post-stroke cognitive impairment [41].

Pre-stroke cognition is assessed by the short version of the Informant Questionnaire on Cognitive Decline in the Elderly (IQCODE). The IQCODE is a validated 16-item questionnaire assessing change in cognition over the course of 10 years [42]. It assesses changes in working, short-term and long-term memory, learning and attention and the ability to perform daily activities. The questionnaire is completed by the nearest relative either in person or by telephone interview. The respondent is asked to rate the change in the patient’s cognitive ability before the AIS or TIA.

### Questionnaires: well-being, depressive symptoms, and physical activity level

Subjective well-being is assessed by the short 5-item World Health Organization Well-Being Index (WHO-5) [43]. Depressive symptoms are measured by the Major Depression Inventory (MDI). The MDI is a 10-item questionnaire covering the symptoms of major depressive disorder [44]. Both the WHO-5 and the MDI are administered at baseline, and at three- and 12-month follow-up. They are answered by patients themselves or with assistance from study personnel.

The Physical Activity Scale for the Elderly (PASE) is used to assess self-reported physical activity level during previous seven days. It is a validated 12-item questionnaire on overall physical activity and it includes work, leisure time-, household- and sports activities to provide a total physical activity score [45]. The PASE is completed by patient interview at baseline and at the follow-up visits.

### Additional assessments

Information about symptom onset, admission time, prior medical history, demographics, alcohol consumption, medication use, admission National Institutes of Health Stroke Scale (NIHSS) score, routine blood chemistry, height, weight, blood pressure, history of smoking, and educational attainment is collected at baseline. Pre-stroke functional ability and post-stroke functional outcome are assessed on the modified Rankin Scale (mRS). Based on information from hospital charts and routine acute MRI, the primary stroke is classified using the Oxfordshire Community Stroke Project classification [46], while the etiological subtype is classified according to the Trial of Org 10,172 in Acute Stroke Treatment (TOAST) criteria [47]. At each follow-up visit, height, weight, and blood pressure are measured. In addition, new clinically verified cardiovascular events are recorded. Cardiovascular events are defined as fatal or non-fatal acute myocardial infarction, AIS, hemorrhagic stroke, or TIA.

### Statistical analysis

The role of capillary dysfunction on PSCI will be assessed by the association between CTH at baseline and PSCI. The primary outcome will be the difference in total RBANS score from three to 12 months follow-up. Secondary outcomes include total RBANS score at 12 months follow-up, difference in MoCA score from three to 12 months follow-up, and MoCA score at 12 months follow-up. All associations will be assessed using linear mixed effects models adjusting for age, sex, educational level, baseline NIHSS, smoking, and diabetes. Analyses will be stratified by APOE genotype (ε4 carriers vs. non-carriers) and pre-stroke cognitive decline (IQCODE > 3.48 vs. IQCODE ≤ 3.48) to assess for effect modification by these factors. Level of cSVD will be assessed as a mediator.

Associations between EV characteristics and cognitive function after stroke will be assessed primarily by exploring differences in EV characteristics at baseline between patients with normal cognition, impaired cognitive function (total RBANS index score 70–85) and reduced cognitive function (total RBANS index score < 70) at 12-month follow-up using analysis of differential expression. Secondary analyses include exploring differences in EV characteristics at baseline among patients with a decline (≥ 6 points) versus patients with no decline in total RBANS score from three to 12 months, between patients with decline (≥ 2 points) versus no decline in MoCA score from three to 12 months follow-up, and between patients above and below MoCA score of 22 and 26 at 12-months follow-up. Analyses will be adjusted for age, sex, educational level, baseline NIHSS, diabetes, and hypertension. In addition, analyses stratified by APOE genotype, pre-stroke cognitive decline, and level of cSVD will be performed.

### Sample size calculation

A previous study could show a correlation between capillary dysfunction and cognitive status in 32 patients with Alzheimer’s disease [48]. To find a similar effect size in a linear model with 10 predictor variables, a sample size of 71 patients is needed (90% power, significance level 0.05). This calculation was performed using the pwr-package in *R* version 4.3.2 [49]. Previous studies of EV characteristics were able to distinguish between stroke severity, lesion volume and outcome when comparing groups of 21 and 20 stroke patients [50]. A sample size of 100 patients with complete follow-up is therefore considered adequate.

## Discussion

With the ENIGMA study we aim to study vascular contributions to cognitive decline and dementia. Using advanced MRI techniques and studying EV characteristics, we aim to investigate capillary dysfunction and EV characteristics as predictors of cognitive decline and cognitive function one year after AIS and TIA.

It is well established that ischemic stroke and cSVD increase risk of cognitive impairment and dementia [5, 51]. However, mechanisms behind vascular cognitive impairment are largely unknown. For this reason, numerous prospective observational studies are examining predictors of post-stroke cognitive decline [52–58] and consortia have been established to drive the research into vascular cognitive impairment forward [59, 60]. With ENIGMA we hope to provide novel knowledge about the microvascular mechanisms behind PSCI and the potential of EVs as biomarkers of the vascular brain reserve and consequently post-stroke cognitive function.

Traditionally, we consider stroke and TIA flow-limiting conditions. However, increasing evidence suggests that we should also consider dysfunction of the capillaries, primarily limiting oxygen extraction rather than blood supply, as an important mechanism of cerebrovascular disease [20, 61]. Capillary dysfunction has been shown to contribute to tissue injury after acute stroke and to cognitive impairment in both vascular dementia and Alzheimer’s disease [21, 48, 61, 62]. New imaging methods, developed by researchers in our group, now enable us to characterize microvascular flow patterns on MRI with the potential to detect and quantify the preclinical manifestations of this contribution to cerebrovascular disease [22, 24]. In addition, advanced diffusion-weighted MRI methods such as diffusion kurtosis imaging may contribute to a better understanding of the microstructure and functional integrity of brain tissue.

EVs may represent biomarkers indicating the current cerebrovascular disease state [27]. The diagnostic and prognostic use of EVs is appealing as they have unique surface markers. The release, content, and composition of these markers are altered during disease in the central nervous system. Further, these changes are detectable in the blood as EVs naturally cross the blood brain barrier [63]. The EV-array technology makes it possible to detect up to 60 different EV surface markers directly from plasma using antibodies as detection agents [38]. EVs derived from e.g. endothelial cells and leucocytes could be of particular interest, as they may reflect vascular dysfunction and contribute to post-stroke neuroinflammation and PSCI. In addition, profiling EV content, such as microRNAs and proteins, can be done using molecular biological techniques including proteomics and next generation sequencing. Specifically, miRNA upregulated in cSVD would be of interest. EVs thus represent a novel reservoir of potential biomarkers of stroke outcomes.

Study results will be published in peer-reviewed journals and reported according to STROBE guidelines. The results will be generalizable to most stroke populations. However, as patients aged < 60 years, patients with known neurodegenerative disease, and patients with pre-stroke mRS > 2 were excluded, the study might not elucidate the associations between PSCI and capillary dysfunction or EV characteristics in patients < 60 years and in patients with more severe pre-stroke disability. Further, although the RBANS has the advantage of different versions for repeated testing, it may not sufficiently test executive function and psychomotor speed, which are cognitive functions often affected in PSCI. Different additional tests could have been performed, e.g. the Trail Making Test (TMT). As this test cannot be scored if the participant takes more than 5 min to complete, the MoCA test, which, among other subtests, contains a short version of the TMT-B was chosen to be performed at baseline as well as at 3- and 12-months follow-up.

Summary and conclusions.

In conclusion, the ENIGMA study is a single-centre prospective clinical observational study with the aim to investigate vascular contributions to cognitive decline and dementia. By studying MRI-defined capillary dysfunction and EV characteristics, the ENIGMA study links novel imaging and basic research techniques to a clinical cohort of stroke patients. With this study we hope to enhance the understanding of the mechanisms behind post-stroke cognitive decline and dementia.

## Data Availability

All investigators will have access to the entire dataset. Trial metadata, including, but not limited to codebooks, data dictionaries and analysis code will be shared on a repository under a permissive license. Upon completing a collaboration agreement, individual patient trial data may be shared upon reasonable request. Patient data will not be made publicly available.

## Abbreviations

AIS: acute ischemic stroke
APOE: apolipoprotein E
BP: blood pressure
cSVD: cerebral small vessel disease
CTH: capillary transit time heterogeneity
DWI: diffusion-weighted imaging
eGFR: estimated glomerular filtration rate
EVs: extracellular vesicles
FLAIR: fluid-attenuated inversion recovery
GRE: gradient echo
IQCODE: informant Questionnaire on Cognitive Decline in the Elderly
MDI: Major Depression Inventory
MoCA: Montreal Cognitive Assessment
MRI: magnetic resonance imaging
mRS: modified Rankin Scale
NIHSS: National Institute of Health Stroke Scale
OCSP: Oxfordshire Community Stroke Project Classification
PASE: Physical Activity Scale for the Elderly
PSCI: post-stroke cognitive impairment
RBANS: Repeatable Battery for the Assessment of Neuropsychological Status
SE: spin echo
STRIVE-1: Standards for Reporting Vascular Changes on Neuroimaging-1
SWI: susceptibility weighted imaging
TIA: transient ischemic attack
TOAST: trial of Org. 10172 in Acute Stroke Treatment
WHO-5: World Health Organization Five Well-Being Index
